# Key problems of the deep Earth

**DOI:** 10.1093/nsr/nwab020

**Published:** 2021-02-10

**Authors:** Ho-Kwang Mao

During Earth's 4.6-billion-year history, its surface has experienced environmental changes that drastically impacted habitability. The changes have been mostly attributed to near-surface processes or astronomical events with little consideration of Earth's deep interior. Recent progresses in high-pressure geochemistry and geophysics, however, indicated that the deep Earth processes may have played a dominant role in the surface habitability [1]. This Special Topic collects a highlight, five perspectives, two research articles and two news articles to address key issues of this paradigm change.

Oxygen, the most abundant element in Earth, is the key element that enables lifeforms to thrive and creates the unique living planet as we know. Recent discoveries on the pressure-altered chemistry revealed that the oxygen cycles through Earth's deep interior could also be the origin of the dynamic mantle processes that dictate the disruptive evolution of the life on surface. The Earth shows a vertical gradient of oxygen fugacity over 10 orders of magnitude from the highly oxidized crust at ambient conditions to the highly reduced core–mantle boundary (CMB) at ∼2900 km depth and 130 GPa. The dynamic process of subducting slab, on the other hand, injects the oxidized crust down into the reduced deep lower mantle. Here, H_2_O is the key compound that acts as a strong oxidant and oxygen transporter. In this Special Topic, Katsura and Fei [2] present a perspective of H_2_O in the asthenosphere, and Walter [3] discusses the H_2_O transport all the way down to the CMB. It has been established that when H_2_O meets Fe at the CMB, Fe can be oxidized to produce superoxide FeO_2_ with pyrite structure. At the middle of the lower mantle (1800 km, 75 GPa), Hu *et al.* [4] show that H_2_O can also react with ferropericlase (Mg, Fe)O, a major lower mantle mineral, to form pyrite-structured superoxide. At shallower depth of 1000 km (40 GPa), Liu *et al.* [5] found that H_2_O induced oxidation of ferropericlase to form Fe_2_O_3+δ_ with a new hexagonal structure. Both reactions result in further oxygen enrichment beyond the already highly oxidized slab materials in the deep mantle environment. The subducting process may compile substantial oxygen-rich materials to form oxygen reservoirs at the CMB.

Recognition of novel materials in deep Earth relies on seismological observations of their characteristic elastic signatures that must be determined *insitu* at high pressure–temperature conditions of the deep interior. Mao *et al.* [6] present recent advances in experimental and theoretical studies of elastic properties of lower mantle minerals. Chen [7] uses longitudinal-wave versus shear-wave velocities’ two-parameter constraint of the observed seismic features to differentiate the superoxides versus partial-melting origins of the ultralow-velocity zone and large low shear velocity province. The oxygen- and iron-rich piles are denser than the surrounding mantle materials and settle stably on top of the core. The CMB is a thermal boundary layer with a very steep temperature gradient controlled by heat transferred from the core [8] that could cause the oxygen reservoirs to melt, lose Fe to the core and leave lighter oxidized component trapped by the overlaying mantle. When a significant quantity of the trapped oxidized component bursts out and ascends, it forms superplume and provides a powerful driving force for chemical convection of the mantle. The oxygen outburst, therefore, could well crank start the plate tectonics of the mantle, and cause splitting and merging of supercontinent. Lin and van Westrenen [9] highlight the significance of extra oxygen in lowering the melting point of rocks and generating large igneous province (LIP). Overall, the oxygen activities in the deep interior could be the common thread for most catastrophic events, including supercontinent cycles, LIPs, atmospheric oxygen fluctuations, snowball Earth and mass extinctions.
